# Targeting Lymph Nodes for Systemic Immunosuppression Using Cell‐Free‐DNA‐Scavenging And cGAS‐Inhibiting Nanomedicine‐In‐Hydrogel for Rheumatoid Arthritis Immunotherapy

**DOI:** 10.1002/advs.202302575

**Published:** 2023-07-12

**Authors:** Furong Cheng, Ting Su, Yangtengyu Liu, Shurong Zhou, Jialong Qi, Weisheng Guo, Guizhi Zhu

**Affiliations:** ^1^ State Key Laboratory of Oncogenes and Related Genes Shanghai Cancer Institute Ren Ji Hospital School of Medicine Shanghai Jiao Tong University Shanghai 200032 China; ^2^ Department of Pharmaceutics and Center for Pharmaceutical Engineering and Sciences School of Pharmacy The Developmental Therapeutics Program, Massey Cancer Center. Virginia Commonwealth University Richmond VA 23298 USA; ^3^ Translational Medicine Center The Second Affiliated Hospital Guangzhou Medical University Guangzhou 510260 China; ^4^ Department of Rheumatology and Immunology Xiangya Hospital Central South University Changsha 410008 China; ^5^ Department of Pharmaceutical Sciences College of Pharmacy Biointerfaces Institute University of Michigan Ann Arbor MI 48109 USA

**Keywords:** cell‐free DNA scavenging, cGAS inhibition, immunotherapy, lymph node targeting, nanoparticle‐in‐hydrogels, rheumatoid arthritis

## Abstract

Rheumatoid arthritis (RA) is a systemic autoimmune disease with pathogenic inflammation caused partly by excessive cell‐free DNA (cfDNA). Specifically, cfDNA is internalized into immune cells, such as macrophages in lymphoid tissues and joints, and activates pattern recognition receptors, including cyclic guanosine monophosphate–adenosine monophosphate synthase (cGAS), resulting in overly strong proinflammation. Here, nanomedicine‐in‐hydrogel (NiH) is reported that co‐delivers cGAS inhibitor RU.521 (RU) and cfDNA‐scavenging cationic nanoparticles (cNPs) to draining lymph nodes (LNs) for systemic immunosuppression in RA therapy. Upon subcutaneous injection, NiH prolongs LN retention of RU and cNPs, which pharmacologically inhibit cGAS and scavenged cfDNA, respectively, to inhibit proinflammation. NiH elicits systemic immunosuppression, repolarizes macrophages, increases fractions of immunosuppressive cells, and decreases fractions of CD4^+^ T cells and T helper 17 cells. Such skewed immune milieu allows NiH to significantly inhibit RA progression in collagen‐induced arthritis mice. These studies underscore the great potential of NiH for RA immunotherapy.

## Graphical abstract

Nanomedicine‐in‐hydrogel (NiH) that co‐delivers cfDNA‐scavenging cationic nanoparticles and a cyclic guanosine monophosphate–adenosine monophosphate synthase inhibitor to draining lymph nodes can decrease the fractions of systemic CD4^+^ T cells and Th17 cells, while increasing systemic immunosuppressive Tregs, MDSCs, as well as M2/M1‐like macrophage ratios. The ability of NiH to elicit systemic immunosuppression and restored immune homeostasis in collagen‐induced arthritis mice suggests that NiH holds the potential to benefit rheumatoid arthritis immunotherapy.

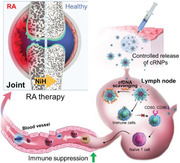

## 1 Introduction

Rheumatoid arthritis (RA) is a chronic autoimmune disease resulting from defective immune tolerance.^[^ ^1^, ^2^ ^]^ While RA pathogenesis remains to be fully understood, the hallmark of RA is the breakdown of immune tolerance that triggers autoantibody production and promotes the infiltration of innate and adaptive immune cells into the synovial membrane.^[^ ^3^, ^4^, ^5^ ^]^ These altogether result in chronic synovitis in joints as well as systemic immune complications.^[^ ^1^, ^2^, ^6^ ^]^ Current RA treatments (e.g., disease‐modifying anti‐rheumatic drugs, non‐steroidal anti‐inflammatory agents, and corticosteroids) are limited by their short‐term alleviation of joint pain and inflammation, and long‐term use of these medications often lead to suboptimal therapeutic response, systemic cumulative toxicity, and pathogenic infection.^[^ ^7^, ^8^, ^9^, ^10^, ^11^ ^]^ This calls for innovative approaches to effective and safe long‐term RA therapy.

Recently, the abundant cell‐free DNA (cfDNA) in peripheral blood and synovial fluid has been shown to be associated with RA pathogenesis.^[^ ^12^, ^13^ ^]^ Specifically, cfDNA is taken up into immune cells such as macrophages, where cfDNA activates a series of pattern recognition receptors (PRRs), including cytosolic double‐stranded DNA (dsDNA) sensor cyclic guanosine monophosphate‐adenosine monophosphate synthase (cGAS) and unmethylated CpG‐rich DNA sensor Toll‐like receptor 9 (TLR9), and thereby enhancing inflammatory responses.^[^ ^12^, ^14^, ^15^, ^16^ ^]^ cGAS activation arouses the aberrant activation of the stimulator of the interferon genes (STING) pathway, which promotes type I interferon (IFN) response.^[^ ^15^ ^]^ Meanwhile, TLR9 activation also enhances proinflammatory responses.^[^ ^16^, ^17^ ^]^ Taken together, cfDNA leads to overly strong systemic inflammatory responses, which promote systemic autoimmunity and contribute to RA progression.^[^ ^12^, ^18^ ^]^ Therefore, systemic cfDNA scavenging and cGAS inhibition represent a promising therapeutic strategy to restrain rheumatoid synovial aggression. Towards this end, current approaches include systemic administration of cfDNA‐scavenging cationic biomaterials, which, however, are associated with concerns over the toxicity caused by the nonspecific cationic biomaterials.^[^ ^19^, ^20^, ^21^ ^]^

Lymph nodes (LNs) are secondary lymphoid tissues that orchestrate a wide range of immune responses and maintain immune homeostasis.^[^ ^22^, ^23^ ^]^ Clinical studies indicated that 82% of RA patients show symptoms of lymphadenomegaly (enlarged LNs), which is associated with RA severity and treatment responsiveness.^[^ ^24^, ^25^, ^26^ ^]^ Moreover, RA human patients have an increased risk to develop lymphoma, which, implies that RA patients may have lost immune homeostasis in LNs, albeit the underlying mechanistic correlation between RA and lymphadenopathy remains to be fully understood.^[^ ^27^, ^28^, ^29^ ^]^ Therefore, we hypothesize that regulating LN immune milieu may elicit systemic rheumatoid immune tolerance, which has been rarely explored for RA treatment.

**Scheme 1:**
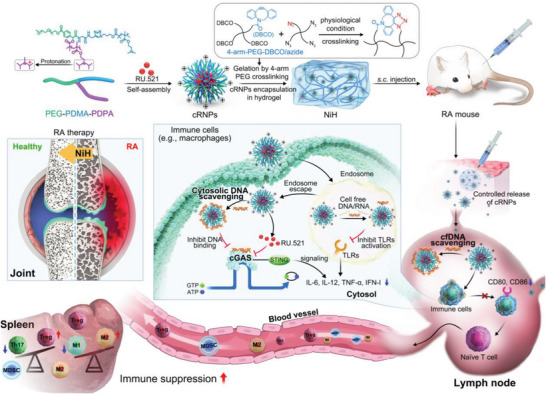
NiH co‐delivered cfDNA‐scavenging cNPs and cGAS inhibitors to draining LNs for systemic immunosuppression in RA immunotherapy. NiH is comprised of injectable hydrogel encapsulated with RU‐loaded cfDNA‐scavenging cNPs (i.e., cRNPs). The hydrogel was formed by the crosslinking of 4‐arm PEG‐dibenzocyclooctyne (4‐arm‐PEG‐DBCO) and 4‐arm PEG‐azide (4‐arm‐PEG‐N_3_) via copper‐free, strain‐promoted azide–alkyne cycloaddition click chemistry. cRNPs were loaded in PEG hydrogel by mixing cRNPs with PEG precursors before cross‐linking. Upon s.c. injection, NiH prolonged the retention of cRNPs in draining LNs, where they concurrently scavenged cfDNA and pharmacologically inhibited cGAS. As a result, NiH suppressed intranodal and systemic inflammation, as evidenced by repolarized macrophages, increased fractions of immunosuppressive cells, and decreased fractions of CD4^+^ T cells and Th17 cells. Such skewed immune tolerance allowed NiH to inhibit RA progression in CIA mice.

Here, we report a nanomedicine‐in‐hydrogel (NiH) composite that co‐delivered cfDNA‐scavenging cationic nanoparticles (cNPs) and a cGAS inhibitor to drain LNs to suppress systemic inflammation for RA treatment (**Scheme** **1**). We first confirmed lymphadenomegaly and elevated systemic inflammation in RA human patients and a RA mouse model. We then investigated the LN immune milieu in RA mice, and showed that these LNs presented an RA‐associated immune signature with elevated levels of cfDNA and cGAS. To suppress the overly strong inflammation in LNs and systemically, we developed NiH that concurrently scavenges cfDNA and inhibit cGAS in immune cells. Specifically, we synthesized and screened a series of cNPs that were loaded with a potent cGAS inhibitor, RU.521 (RU). The resulting RU‐loaded cNPs (cRNPs) were further formulated into an injectable hydrogel, which allowed efficient homing and retention of cRNPs in draining LNs upon subcutaneous (s.c.) injection using syringe needles. In LNs, peripheral blood, and spleens of collagen‐induced arthritis (CIA) mice, NiH repolarized macrophages and expanded the populations of immunosuppressive regulatory T cells (Treg) and myeloid‐derived suppressor cells (MDSCs), while decreasing the fractions of CD4^+^ T cells and T helper 17 (Th17) cells. As a result, NiH efficiently restrained RA progression in CIA mice. These results underscore the potential of LN and systemic immunosuppression using NiH for RA immunotherapy.

## 2 Results and Discussion

### 2.1 The lymphoid Tissues of CIA Mice and Human RA Patients are Highly Proinflammatory with Abundant cfDNA and Upregulated cGAS

**Figure 1:**
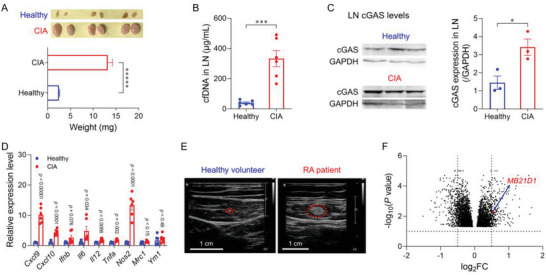
The LNs of CIA mice and human RA patients are highly proinflammatory with abundant cfDNA and upregulated cGAS. A) Photographs and weights of LNs from healthy mice and CIA mice. B) cfDNA levels in LNs from healthy mice and CIA mice. C) Western blot results of cGAS levels in LNs from healthy mice and CIA mice. GAPDH: glyceraldehyde 3‐phosphate dehydrogenase. D) qPCR results for a panel of cytokine and chemokine gene transcripts in LNs from healthy mice and CIA mice. E) Representative ultrasound images of LNs from healthy humans and RA human patients (red circles denote LNs). F) Volcano plots of bulk RNA‐seq analysis of genes differentially expressed in PBMCs of RA patients and healthy humans (*p* < 0.05).^[^ ^39^ ^]^ Data: mean ± s.e.m. *n* = 3. *p*‐Values were determined by one‐way (C) or two‐way (A, B, D) ANOVA, followed by Tukey's multiple comparison test (*p < 0.05; ***p < 0.001; *****p < 0.00001).

RA is a systemic autoimmune disease with significant lymphadenopathy.^[^ ^1^, ^24^ ^]^ We hypothesized that RA patients have abundant cfDNA in lymphoid tissues, such as LNs and spleens, which causes overly strong intranodal and systemic inflammation by cfDNA activation of PRRs such as cytosolic DNA sensor cGAS.^[^ ^15^, ^17^, ^25^ ^]^ cGAS activation by DNA results in proinflammatory type I IFN (IFN‐I) responses, which were shown to cause arthritis in three prime repair exonuclease 1 knockout (*Trex1^−/−^*) and *DNaseII^−/−^* mice, verifying a key role of cGAS in RA development.^[^ ^30^, ^31^ ^]^ We first tested the above hypothesis in CIA mice, a commonly used RA mouse model. We harvested LNs from CIA mice, with age‐matched healthy mice as controls, to analyze LN levels of cfDNA and cGAS. CIA mice showed significant lymphadenomegaly, with a 4.3‐fold average weight of CIA mouse LNs relative to healthy mouse LNs (**Figure** **1**A). Further, CIA mouse LNs exhibited 7.4‐fold increase of cfDNA relative to that of the healthy mouse LNs (*p* = 0.00027) (Figure 1B). Western blot showed 2.4‐fold cGAS protein level in CIA mouse LNs, relative to healthy mouse LNs (Figure 1C). Such elevated cfDNA and cGAS protein levels in CIA mouse LNs are expected to promote proinflammatory responses.

The LN immune environment of RA patients continually evolves during RA progression.^[^ ^25^, ^32^, ^33^, ^34^ ^]^ We analyzed the cellular and molecular immune environment in CIA mouse LNs by flow cytometry and quantitative polymerase chain reaction (qPCR). CIA mouse LNs showed elevated fractions of macrophages (Mφ: 0.38%) and dendritic cells (DCs: 1.62%) among CD45^+^ cells relative to those of healthy mouse LNs (Mφ: 0.17%; DCs: 1.09%) (Figure S1, Supporting Information). Meanwhile, the percentage of immunosuppressive Treg cells was significantly decreased in LNs compared with the healthy group (Figure S1, Supporting Information). As shown by qPCR analysis of the transcript levels of key immune markers, CIA mouse LNs exhibited notably enhanced expression of proinflammatory genes (*Ifnb*, *Tnfa*, *Il6*, *Il12*, *Nos2*) that are often associated with M1‐like macrophages, in contrast to immunosuppressive M2‐like macrophage‐associated genes (*Mrc1* and *Ym1*) (Figure 1D). Moreover, CIA mouse LNs also showed elevated gene transcript levels of chemokines *Cxcl9* (9.1‐fold increase) and *Cxcl10* (3.1‐fold increase), relative to healthy mouse LNs. Importantly, the spleen of CIA mice exhibited consistent immune microenvironments relative to that in LNs (Figure S2, Supporting Information), which verified the systemic pathogenic inflammation in CIA mice. Overall, these studies demonstrate that, relative to healthy mice, CIA mouse LNs and spleen exhibited upregulated cGAS expression, elevated cfDNA level, and immune imbalance.

Consistently, in RA human patients, we verified significant lymphadenopathy (Figure 1E; Figure S3, Supporting Information). The mean area of LNs in RA human patients was 20‐fold that of healthy volunteers (Figure S4, Supporting Information). Further, RNA sequencing (RNA‐seq) analysis of human peripheral blood mononuclear cells (PBMCs) revealed that cGAS transcript levels (Mab‐21 domain‐containing 1 or *MB21D1*) increased significantly in RA human patients, relative to healthy humans (*p* < 0.01) (Figure 1F). The lymphatic system in RA patients drains synovial fluid from inflamed synovium, where abundant cfDNA can activate PRRs to elicit inflammatory responses.^[^ ^25^, ^35^, ^36^, ^37^, ^38^ ^]^ Taken together, these results build the foundation to suppress systemic RA‐associated inflammation via cfDNA scavenging and cGAS inhibition in LNs for RA therapy.

### 2.2 cNPs Scavenged DNA by Electrostatic Interactions and Inhibited DNA‐Elicited Proinflammatory Responses in Macrophages

**Figure 2:**
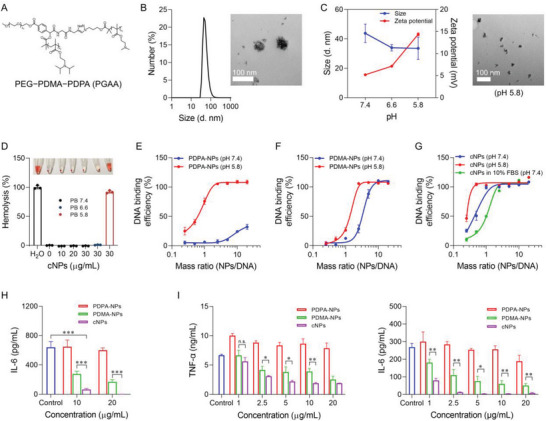
cNPs scavenged extracellular and intracellular DNA to inhibit proinflammatory responses in macrophages. A) Chemical structure of cationic polymer PGAA. B) DLS results of the hydrodynamic diameters (left) and a TEM image (right) of cNPs. C) Hydrodynamic sizes and zeta potential of cNPs at different pH conditions (left) and a TEM image of cNPs at pH 5.8 (right). D) Hemolysis assay results showing that cNPs caused pH‐dependent erythrocyte membrane destabilization. E–G) DNA binding by PDPA‐NPs (E), PDMA‐NPs (F), and cNPs (G) in buffers (pH7.4 or pH5.8), and 10% fetal bovine serum (FBS) at 37°C. H) Enzyme linked immunosorbent assay (ELISA) results showing that cNPs inhibited IL‐6 production in RAW264.7 macrophages. RAW264.7 cells were co‐incubated with cNPs and 1 µm CpG at 37°C for 24 h, followed by ELISA measurement of medium IL‐6 concentrations. I) cNPs efficiently inhibited TNF‐α and IL‐6 secretion in RAW264.7 cells pretreated with CpG in a dose‐dependent manner. RAW 264.7 cells were first incubated with 1 µM CpG at 37°C for 4 h, washed, and then replenished with fresh media containing cNPs. After 24 h, medium concentrations of TNF‐α and IL‐6 were measured by ELISA. *n* = 3. Data: mean ± SD. *p*‐Values were determined by two‐way ANOVA followed by Tukey's multiple comparison test (*n.s*.: not significant; *p < 0.05; **p < 0.01; ***p < 0.001).

Given the high cfDNA levels and upregulated cGAS in the lymphoid tissues that in part contribute to imbalanced proinflammation in RA patients, we attempted to develop cGAS‐inhibitor‐loaded cNPs that scavenge cfDNA and pharmacologically inhibit cGAS activation as a novel strategy for RA treatment. As we previously reported,^[^ ^40^ ^]^ we synthesized cationic miktoarm star polymer methoxy poly(ethylene glycol)‐poly(2‐(dimethylamino)ethyl methacrylate)‐poly(2‐(diisopropylamino)ethyl methacrylate) (PEG−PDMA−PDPA or PGAA) with 46 PDMA repeat units and 9 PDPA repeat units per polymer on average (**Figure** **2**A). PGAA synthesis was verified by ^1^H NMR (Figure S5, Supporting Information), with a molecular weight (*M*_w_) of 13.3 kDa as determined by gel permeation chromatography (GPC) (Figure S6, Supporting Information). At pH 7.4, amphiphilic PGAA was self‐assembled into multivesicular micellular cNPs with hydrodynamic diameters of ≈51 nm as shown by dynamic light scattering (DLS) and transmission electron microscopy (TEM) (Figure 2B), +5.2 mV of zeta potential, and good stability (Figure S7, Supporting Information). Due to the protonation of PDPA at relatively low pH, cNPs exhibited a pH‐responsive reduction of hydrodynamic NP sizes (≈30 nm in diameter at pH 5.8) and a pH‐responsive increase of zeta potentials (+14.4 mV at pH 5.8) (Figure 2C). Further, cNPs showed enhanced erythrocyte membrane destabilization at acidic conditions, suggesting the potential of cNPs for efficient escape from acidic endosome that would be pivotal for cGAS inhibitor to reach its target cytosolic cGAS (Figure 2D).

The electrostatic charge of cNPs determines their DNA binding capacity. We first evaluated the DNA binding ability of cNPs using calf thymus DNA as a model. In PBS (pH 7.4), PEG‐PDMA nanoparticles (PDMA‐NPs) showed a stronger DNA binding ability than did PEG‐PDPA NPs (PDPA‐NPs), due to the positive zeta potential of PDMA‐NPs (Figure 2E,F). Furthermore, the DNA binding ability of PDPA‐NPs dramatically increased at pH 5.8, relative to that at pH 7.4 (Figure 2E), likely due to PDPA protonation. Remarkably, cNPs exhibited higher DNA binding capacity than PDMA‐NPs at <0.25 of NPs/ DNA ratios (Figure 2G and Figure S8, Supporting Information). The presence of serum did not significantly change the DNA‐binding capacity of cNPs with >1 of NPs/DNA ratios, and reduced DNA binding on cNPs with <1 of NPs/DNA ratios (Figure 2G). In addition, at pH 5.8, despite the low NPs/DNA ratio of 0.25, ≈50% DNA was bound by cNPs, indicating pH‐responsive enhancement of DNA binding (Figure 2G).

Given that cNPs can bind with and thus scavenge DNA, we investigated the ability of cNPs to inhibit the proinflammatory responses elicited by extracellular/internalized DNA in immune cells. cNPs demonstrated good biocompatibility at concentrations of up to 50 µg mL^−1^ as shown by MTS (3‐(4,5‐dimethylthiazol‐2‐yl)‐5‐(3‐carboxymethoxyphenyl)‐2‐(4‐sulfophenyl)‐2H‐tetrazolium) assay in RAW264.7 murine macrophages after a treatment for 24 h (Figure S9, Supporting Information). Therefore, we chose concentrations of ≤50 µg mL^−1^ cNPs for further studies in cells. To study whether cNPs attenuated DNA‐mediated inflammation. For extracellular DNA, cNPs and PDMA‐NPs inhibited the ability of CpG oligonucleotide, an immunostimulant TLR9 agonist that was added to cell culture medium at the same time as NPs, to induce the production of proinflammatory cytokines (e.g., interleukin‐6 or IL‐6, tumor necrosis factor‐α or TNF‐α) in RAW264.7 macrophages after treatment for 24 h, in a dose‐dependent manner (Figure 2H and Figure S10, Supporting Information). Specifically, 20 µg mL^−1^ cNPs almost completely inhibited the production of IL‐6 (99.8% reduction) and TNF‐α (99.3% reduction), in contrast to only 73.7% reduction of IL‐6 and 32.2% reduction of TNF‐α by 20 µg mL^−1^ PDMA‐NPs, presumably due to the stronger DNA binding ability of cNPs than PDMA‐NPs. Even 10 µg mL^−1^ cNPs showed nearly 90% inhibition of the above proinflammatory cytokine production (Figure 2H). These results demonstrated the ability of cNPs to scavenge extracellular/internalized immunostimulatory DNA and subsequently inhibit the pro‐inflammatory responses elicited by these DNA in immune cells.

To resemble the clinical setting of RA therapy with abundant pre‐existing DNA, RAW264.7 cells were first treated with CpG for 4 h to allow its cell uptake and PRR activation. These cells were then washed and replenished with fresh cell culture medium to remove extracellular CpG, followed by treatment with cNPs or controls for 24 h. As a result, cNPs significantly reduced TNF‐α and IL‐6 levels in a dose‐dependent manner, which outperformed PDMA‐NPs (Figure 2I). For example, as low as 2.5 µg mL^−1^ cNPs significantly reduced the secretion of TNF‐α (by 54%) and IL‐6 (by 95%). By contrast, 2.5 µg mL^−1^ PDMA‐NPs only showed 37% TNF‐α and 59% IL‐6 concentration reductions. These data clearly demonstrate that cNPs scavenged intracellular CpG to inhibit the resulting proinflammatory response.

### 2.3 cRNPs Scavenged cGAS‐Activating DNA and Deliver cGAS Inhibitors for Efficient cGAS Inhibition

**Figure 3:**
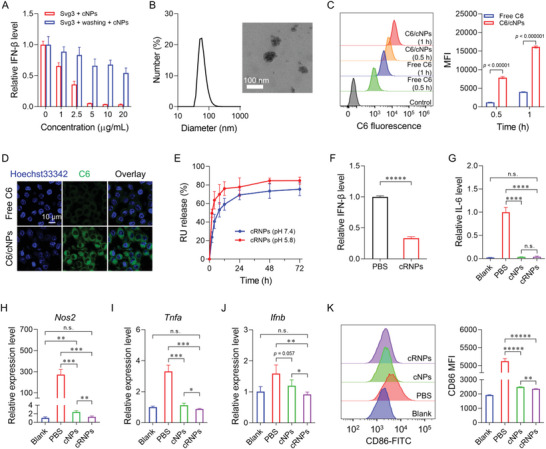
cRNPs inhibited cGAS activation and immunostimulatory DNA‐mediated proinflammatory responses in macrophages. A) ELISA results showing that cNPs inhibited IFN‐β production in RAW264.7 cells pretreated with cGAS‐agonistic Svg3 oligonucleotide. For Svg3 + cNPs, cells were treated with 100 nM Svg3 and a series of concentrations of cNPs for 24 h. For Svg3 + washing + cNPs, cells were first treated with 100 nm Svg3 for 4 h, then washed, and further treated with cNPs for 24 h. Medium IFN‐β concentrations were measured by ELISA. B) Hydrodynamic diameters and a TEM image of cRNPs. C) Representative flow cytometry histogram (left) and mean fluorescence intensity (MFI) (right) of C6 in RAW264.7 cells after incubation with free C6 or C6/cNPs for 0.5 or 1 h. D) Confocal microscopy of the intracellular C6 delivery in RAW264.7 cells (treatment: 1 h). E) In vitro release kinetics of RU from cRNPs. F) ELISA results showing that cRNPs inhibited IFN‐β production in Svg3‐pretreated RAW264.7 cells. Cells were pre‐treated with 100 nM Svg3 for 4 h, then washed and further treated with cRNPs for 24 h (cNPs: 20 µg mL^−1^; RU: 2 µm). Medium IFN‐β concentrations were measured by ELISA. G) cNPs and cRNPs showed complete inhibition of IL‐6 production in RAW264.7 cells pretreated with 1 µm CpG + 100 nm Svg3 for 4 h. Pretreated cells were washed and further treated with cNPs or cRNPs for 24 h. Medium IL‐6 concentrations were measured by ELISA. H–J) qPCR results for proinflammatory cytokine genes in RAW264.7 cells treated with 1 µm CpG, 100 nm Svg3, and cRNPs or controls (RU: 2 µm) for 24 h. K) Representative flow cytometry histogram (left) and MFI (right) of CD86 on RAW264.7 cells treated as in (H–J). DNA was transfected using Lipofectamine 2000. *n* = 3. Data: mean ± SD. *p*‐Values were determined by one‐way (J) or two‐way (C, F‐I, K) ANOVA, followed by Tukey's multiple comparison test (*n.s*.: not significant; *p < 0.05; **p < 0.01; ***p < 0.001; ****p < 0.0001; *****p < 0.00001).

Upon cell uptake, cytosolic cfDNA activates cGAS in a length‐dependent manner for IFN‐I responses that are associated with RA progression. As shown above, cGAS expression was upregulated in RA human patients and CIA mice. To study the ability of cNPs to scavenge cGAS‐activating DNA and inhibit cGAS activation, we used a cGAS‐activating oligonucleotide, Svg3, that we recently developed in the lab (unpublished). When RAW264.7 cells were transfected with Svg3 by Lipofectamine‐2000 and cNPs at the same time for 24 h, cNPs inhibited Svg3‐mediated cGAS activation in a dose‐dependent manner (**Figure** **3**A). Specifically, cNPs resulted in 34% and 64% reduction of IFN‐I at the concentrations of 1 and 2.5 µg mL^−1^, respectively. In parallel, RAW264.7 cells were pre‐treated with Svg3 as above for 4 h, followed by washing cells to remove extracellular Svg3 and cNP treatment for 24 h. cNPs still exhibited significant inhibition of Svg3‐mediated cGAS activation (Figure 3A). Compared with simultaneous treatment with Svg3 and cNPs, Svg3 pretreatment reduced the ability of cNPs to inhibit cGAS activation, likely because of the incomplete scavenging of intracellular Svg3 by cNPs. Overall, these results demonstrate the ability of cNPs to scavenge cGAS‐activating DNA and hence inhibited DNA‐mediated cGAS activation.

To further promote the cGAS inhibition efficacy, we loaded cNPs with a small molecular cGAS inhibitor, RU. The resulting cRNPs had hydrodynamic diameters of approximately 63 nm as shown by DLS and TEM (Figure 3B), with a zeta potential of +6.2 mV in PBS (pH 7.4). Next, we studied the intracellular delivery of hydrophobic RU via cRNPs using small molecular fluorescent Coumarin 6 (C6) to mimic RU. In RAW264.7 cells, C6‐loaded cNPs (C6/cNPs) significantly enhanced the cellular uptake of C6 by 6.7 times relative to free C6 after treatment for 0.5 h, and the cell uptake of C6/cNPs further increased after treatment for 1 h (Figure 3C). Confocal microscopy verified that cNPs promoted the cell uptake of C6 (Figure 3D and Figure S11, Supporting Information). RU release kinetics from cRNPs showed a burst release of RU in the first 4 h (Figure 3E). Moreover, within 12 h, 59% and 76% of RU were released at pH 7.4 and pH 5.8, respectively, indicating pH‐responsive drug release that is expected to facilitate RU release from cRNPs upon cell uptake into acidic endolysosome. Next, we evaluated the ability of cRNPs to inhibit cGAS activation medicated by cGAS‐agonistic Svg3. RAW264.7 macrophages were pretreated with Svg3 for 4 h, and cells were then washed to remove extracellular Svg3, followed by treatment with cRNPs for 24 h. As a result, cRNPs significantly inhibited the ability of Svg3 to activate cGAS for IFN‐I responses. Specifically, cRNPs inhibited IFN‐β production in Svg3‐treated macrophages by 67% (Figure 3F). Meanwhile, relative to blank cNPs, RU loading in cRNPs did not interfere with the TLR inhibition by cRNPs, as shown by the IL‐6 production in RAW264.7 cells pretreated with both CpG and Svg3 (Figure 3G). This indicates that cRNPs scavenged both CpG and Svg3 while pharmacologically inhibiting cGAS for optimal inhibition of cfDNA‐elicited proinflammatory responses. Overall, these results demonstrate that cRNPs inhibited cGAS activation in the presence of cGAS‐activating DNA.

### 2.4 cRNPs Suppress the Activation of M1‐like Macrophages

M1‐like macrophages play critical roles in RA development by eliciting proinflammatory responses. ^[^ ^41^, ^42^ ^]^ We investigated the impact of cRNPs on M1‐like macrophage activation in the presence of immunostimulatory DNA. RAW264.7 macrophages were treated with CpG + Svg3 together with cRNPs, cNPs, or blank, respectively for 24 h. As shown by qPCR, both cNPs and cRNPs inhibited the expression of proinflammatory M1‐like macrophage‐associated genes (*Tnfa*, *Ifnb*, *Nos2*; Figure 3H–J). Remarkably, cRNPs outperformed cNPs and completely inhibited the expression of these proinflammatory cytokine genes. This suggests that the dual mechanisms of cfDNA scavenging and pharmacological cGAS inhibition in cRNPs contributed to the optimal inhibition of proinflammation. Consistently, flow cytometric analysis of RAW264.7 macrophages treated as above revealed that cRNPs significantly inhibited the expression of M1‐like macrophage markers such as co‐stimulatory factors CD86 (reduction by 86.7%) and CD80 (reduction by 87.3%), and proinflammation‐associated NOS2 (reduction by 52.4%) (Figure 3K and Figure S12, Supporting Information). These results demonstrated the potent inhibition of proinflammation in M1‐like macrophages by cRNPs.

### 2.5 Hydrogel Prolonged the Retention of cRNPs in LNs

**Figure 4:**
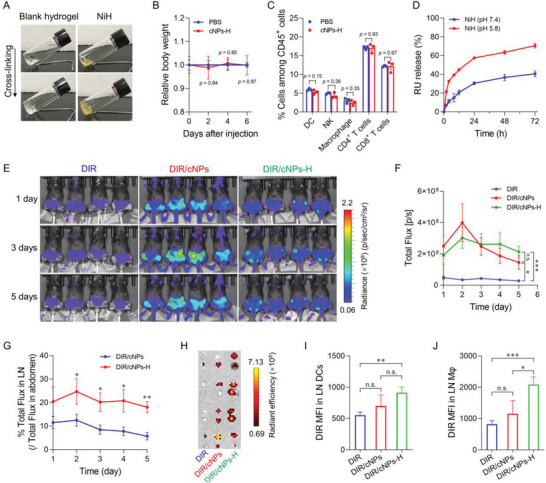
NiH prolonged drug retention in LNs while reducing systemic drug dissemination. A) Gelation of blank hydrogel and NiH as shown by vial tilting. B) Relative mouse body weights after s.c. injection of cNPs‐H and PBS control (cNPs: 40 mg kg^−1^). C) Flow cytometry results showing mouse PBMC immune cell counts 6 days after s.c. injection of cNPs‐H and PBS control. D) In vitro release kinetics of RU from NiH (*n* = 3). E) IVIS images of C57BL/6 mice over 1 – 5 days after s.c. injection of DIR/cNPs‐H and controls (*n* = 4). F) Quantification of DIR fluorescence intensities in the draining inguinal LNs of the above mice. G) The percentage of total fluorescence in LNs relative to that in abdomen from the above mice. H) IVIS images of *ex vivo* LNs from the above mice 5 days after administration. I,J) Flow cytometric analysis of DIR uptake by LN‐residing macrophages and DCs. Data: mean ± SD (D) and mean ± s.e.m. *p*‐Values were determined by one‐way (G, J) or two‐way (B, C, F, I) ANOVA, followed by Tukey's multiple comparison test (*n.s*.: not significant; *p < 0.05; **p < 0.01; ***p < 0.001).

As shown above, likely because the PEGylation of cNPs shielded the excessive positive charge on NP surfaces, cNPs showed good biocompatibility in vitro and in vivo (Figures S9 and S13, Supporting Information). Nonetheless, cationic biomaterials are often associated with nonspecific electrostatic interactions with various biomolecules and cells, leading to suboptimal biosafety and representing a hurdle for their clinical applications. Hydrogel has great potential for sustained drug release delivery while decreasing adverse side effects resulting from rapid and random drug dissemination.^[^ ^43^ ^]^ To achieve sustained drug release and shielding cationic charges of cRNPs from non‐specific biointeractions, we employed a biocompatible PEG hydrogel to develop an injectable nanomedicine (cRNPs)‐in‐hydrogel composites NiH for RA therapy. Specifically, PEG hydrogel was formed by the crosslinking of 4‐arm‐PEG‐DBCO and 4‐arm‐PEG‐N_3_ via copper‐free, strain‐promoted azide–alkyne cycloaddition click chemistry. cRNPs were loaded in PEG hydrogel by mixing cRNPs with PEG precursors before cross‐linking. NiH (4%) underwent rapid solution‐to‐gel (sol‐to‐gel) transformation within 3 min, making it suitable for syringe injection, and cRNPs encapsulation in hydrogel did not interfere with the gelation (**Figure** **4**A). Furthermore, upon s.c. injection using syringe needles, cNPs‐in‐hydrogel (cNPs‐H) did not cause any significant mouse body weight loss and pathological changes in major organs, nor did it affect the cell densities of PBMC DCs, macrophages, and lymphocytes (Figure 4B,C; Figure S14, Supporting Information). NiH mediated a controlled drug release, with 31% and 58% RU released from NiH at pH 7.4 and pH 5.8, respectively, within 24 h, and took longer to release equivalent amounts of drugs compared with cRNPs (Figures 3E and 4D). This indicates the controlled drug release from nanomedicine‐in‐hydrogel composites.

Targeted drug delivery into immunomodulatory tissues and cells, such as LNs and intranodal macrophages that play important roles in RA development,^[^ ^25^, ^44^ ^]^ are desired for optimal immunomodulatory efficacy and RA therapy with minimal systemic toxicity caused by rapid systemic dissemination. To this end, DIR was used as a model fluorescent probe to evaluate the ability of DIR‐loaded cNPs‐in‐hydrogel (DIR/cNPs‐H) for drug delivery to LNs. DIR/cNPs‐H and controls were s.c. injected at mouse tail base. IVIS imaging of DIR fluorescence in mice showed that, relative to free DIR, DIR/cNPs and DIR/cNPs‐H significantly enhanced DIR accumulation in draining LNs 1 – 5 days after administration (Figure 4E,F). Specifically, relative to DIR/cNPs that showed rapid reduction of DIR fluorescence intensity after 2 days, DIR/cNPs‐H prolonged DIR retention in draining LNs with 1.47‐ and 7.8‐fold LN retention on day 5 relative to DIR/cNPs and free DIR, respectively. Consistently, on day 5, relative to DIR/cNPs, DIR/cNPs‐H showed a 217% increase of DIR fluorescence intensity ratios in LNs over that in abdomen, indicating efficient drug delivery and retention in LNs as well as reduced systemic drug dissemination by cNPs‐H (Figure 4G). On day 5, ex vivo imaging of resected LNs verified the efficient DIR retention in LNs by DIR/cNPs‐H (Figure 4H). Further, flow cytometric analysis of LN‐residing single cells showed that DIR was efficiently taken up by LN‐residing macrophages and DCs, providing the basis for the targeting and immunomodulation of these cells by cRNPs (Figure 4I,J). Main organs were harvested 5 days after s.c. administration of DIR, DIR/cNPs, and encapsulated DIR/cNPs in hydrogel (DIR/cNPs‐H), respectively, for ex vivo IVIS imaging. While free DIR was rapidly cleared out of the body and DIR/cNPs showed high accumulation in the liver and lung, DIR/cNPs‐H reduced the accumulation of DIR/cNPs in these healthy organs. Combined with the efficient accumulation of cNPs‐H in draining lymph nodes, these results demonstrate the potential of nanomedicine‐in‐hydrogel to reduce drug random (systemic) dissemination, and thereby ameliorating adverse side effects (Figure S15, Supporting Information). Collectively, these results demonstrate the ability of NiH for controlled drug release and targeted drug delivery into immunomodulatory tissues and cells while reducing unwanted systemic dissemination.

### 2.6 Therapeutic Efficacy of NiH in CIA Mice

**Figure 5:**
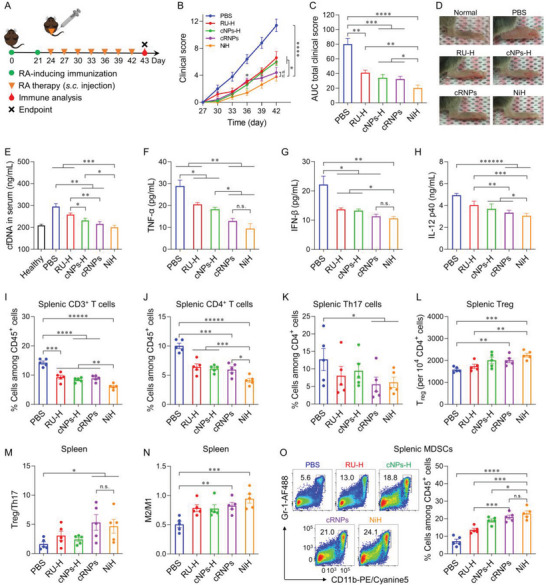
NiH inhibited RA progression in CIA mice via systemic cfDNA scavenging and immune suppression. A) Experiment design. DBA/1J mice were immunized at the end of the tail on day 0 and day 21. On day 24, mice started to be treated s.c. with NiH or controls every 3 days until day 43, when PBMCs and organs were collected for analysis. B, C) Total clinical score curves (B) and their AUCs (C) of paws. D) Representative photographs of hind paw from as‐treated mice on day 43. E) Serum cfDNA levels in as‐treated mice on day 43. F–H) Serum levels of TNF‐α (F), IFN‐β (G), and IL‐12 p40 (H) in as‐treated mice on day 43. I–O) Analysis of splenocytes from as‐treated mice on day 43. I–K) The fractions of splenic CD3^+^ T cells (I), CD4^+^ T cells (J), and Th17 cells (K) among CD45^+^ cells from as‐treated mice on day 43. L) Treg fractions among CD45^+^ cells from as‐treated mice on day 43. M–O) NiH enhanced Treg/Th17 ratio (M), M2/M1‐like macrophage ratio (N), and the fractions of splenic MDSCs (O), indicating the enhanced systemic immunosuppression. *n* = 5. Data: mean ± s.e.m. *p*‐Values were determined by one‐way (B, C, E, G, H, K, N) or two‐way (F, I, J, L, M, O) ANOVA, followed by Tukey's multiple comparison test (*n.s*.: not significant; *p < 0.05; **p < 0.01; ***p < 0.001; ****p < 0.0001; *****p < 0.00001; ******p < 0.000001).

Next, we evaluated the therapeutic efficacy of NiH in CIA mice (**Figure** **5**A). CIA mice were treated with NiH by s.c. injection at the tail base every 3 days, followed by clinical scoring to evaluate the therapeutic efficacy. While both RU‐loaded hydrogel (RU‐H) (*p* = 0.0064) and cNPs‐H (*p* = 0.001) exhibited significantly reduced swelling in paws, relative to PBS; cRNPs outperformed the therapeutic efficacy of RU‐H and cNPs‐H and remarkably reduced arthritis severity compared to RU‐H (*p* = 0.02) and cNPs‐H (*p* = 0.017; Figure 5B). Consistently, NiH reduced the area under the curve (AUC) of clinical scores and mouse paw thickness, relative to the above controls (Figure 5C,D). The superior therapeutic efficacy of NiH is likely attributed to its controlled drug release, targeted drug delivery, and prolonged drug retention in lymphoid tissues and immunomodulatory cells.

RA development is associated with abundant cfDNA and strong proinflammatory responses, not only in peripheral lymphoid tissues such as LNs, but also in systemic blood circulation and associated compartments.^[^ ^6^, ^12^, ^18^, ^45^ ^]^ Therefore, first, we investigated the impact of NiH treatment on the serum levels of cfDNA in CIA mice. Consistent with the in vitro DNA binding ability, on day 43, all cNPs‐containing formulations scavenged serum cfDNA, with 21% (cNPs‐H), 27% (cRNPs), and 32% (NiH) decreases relative to PBS treatment, respectively (Figure 5E). Importantly, relative to PBS, RU‐H, or cNPs‐H, treatment with cRNPs (*p* = 0.29) and NiH (*p* = 0.23) resulted in the reduction of serum cfDNA to basal levels comparable to that in healthy mice. Second, on day 43, we studied the impact of NiH on the serum levels of proinflammatory cytokines in CIA mice. cfDNA can activate macrophages to secrete proinflammatory cytokines which play a fundamental role in RA development. In CIA mice treated as above, NiH as well as cRNPs significantly reduced the serum levels of TNF‐α, IFN‐β, and IL‐12, relative to treatments of PBS, RU‐H, and cNPs‐H (Figure 5F–H).

Next, we assessed the impact of NiH on the frequencies of systemic T cells, including total CD3^+^ T cells, CD4^+^ T cells, CD8^+^ T cells, and Th17 cells, in spleen and peripheral blood in CIA mice (Figure S16, Supporting Information). On day 43, relative to RU‐H, cNPs‐H, and cRNPs, NiH significantly reduced the percentage of CD3^+^ T cells among CD45^+^ cells in spleen and PBMCs (Figure 5I and Figure S17A, Supporting Information). Specifically, NiH reduced CD3^+^ T cell percentages by 57% and 58% in the spleen and PBMCs, respectively. As T cells initiate a serial immune cascade and can extravasate from blood vessels to an inflamed joint in response to proinflammatory cytokines and chemokines, the ability of NiH to reduce the CD3^+^ T cell densities in spleen and peripheral blood may slow RA progression. Due to defects in the DNA repair machinery in RA patients, naïve CD4^+^ T cells transition into highly tissue‐invasive and proinflammatory effector cells, which can be further activated by arthritogenic antigens.^[^ ^2^ ^]^ On day 43, relative to PBS, cRNPs reduced the percentages of CD4^+^ T cells among CD45^+^ cells in the spleen (*p* = 0.00034) and PBMCs (*p* = 0.0043) of CIA mice treated as above (Figure 5J and Figure S17B, Supporting Information). Remarkably, relative to cRNPs, NiH further reduced such fractions of CD4^+^ T cells by 31% in spleen and 4.5% in PBMCs. Moreover, cRNPs and NiH also reduced the percentages of CD8^+^ T cells among CD45^+^ cells in spleen and PBMCs (Figures S17C and S18, Supporting Information). Finally, cRNPs and NiH significantly decreased the percentage of Th17 cells among CD45^+^ cells (Figure 5K). The ability of NiH to reduce the frequency of multiple T cell subsets is likely attributed to cfDNA scavenging and cGAS inhibition. Taken together, these studies demonstrated that NiH efficiently scavenged cfDNA, inhibited proinflammatory responses, and reduced systemic T cell frequencies in CIA mice, all of which are expected to inhibit RA progression.

Finally, we evaluated the impact of NiH on the frequencies of systemic immunosuppressive cell subsets, including Treg, MDSCs, as well as M2/M1 ratio. Treg can inhibit inflammation and promote immune tolerance, but Treg proportion in RA patients is often reduced compared to healthy individuals.^[^ ^46^, ^47^ ^]^ Recovering Treg holds the potential to improve RA therapy. In CIA mice treated as above, relative to PBS treatment, NiH significantly increased the Treg counts in spleen (by 42%) and PBMCs (by 57%), which outperformed cRNPs and other control formulations (Figure 5L and Figure S19A, Supporting Information). Importantly, NiH increased the Treg/Th17 ratio, which is expected to benefit immune balance and immune tolerance (Figure 5M). In addition, relative to PBS, NiH as well as cRNPs increased the M2/M1 ratio, which predicts the immunosuppressive status, in spleen and PBMCs (Figure 5N and Figure S19B, Supporting Information). Finally, compared to PBS, NiH and cRNPs significantly increased the percentages of immunosuppressive MDSCs among CD45^+^ cells (Figure 5O and Figure S20, Supporting Information). Overall, NiH promoted systemic immune suppression and tolerance, which is expected to promote immune homeostasis and eventually benefit RA therapy.

## 3 Discussion

RA is a chronic systemic inflammatory disease with defective immune tolerance.^[^ ^1^ ^]^ LNs, which harbor a variety of immune cells such as macrophages, DCs, and T cells, play a critical role as key orchestrators to maintain immune homeostasis.^[^ ^22^, ^23^ ^]^ The lymphatic systems in RA patients remove synovial fluid containing cfDNA as well as proinflammatory cytokines and cells from inflamed synovium.^[^ ^15^, ^17^, ^25^ ^]^ As a result, cfDNAs can be internalized into and active LN‐residing immune cells via activating PRRs such as cGAS, resulting in enhanced proinflammatory responses.^[^ ^12^, ^14^, ^15^, ^16^ ^]^ Indeed, clinical studies revealed that more than half of RA patients have enlarged LNs, often due to the expanded immune cell populations in LNs.^[^ ^24^, ^25^, ^26^ ^]^ cGAS is an emerging RA therapeutic target that has been shown to contribute to RA development.^[^ ^30^, ^31^ ^]^ We showed that the cGAS gene expression is significantly upregulated in RA patients compared with healthy humans. Consistently, the levels of cfDNA and cGAS in LNs of CIA mice were significantly higher than healthy mice. Moreover, CIA mouse LNs and spleens lost immune balance with reduced immunosuppression relative to healthy counterparts. Inspired by these, we hypothesize that LN‐homing nanoparticles that deliver DNA‐scavenging polymers and cGAS inhibitors can suppress proinflammatory responses and recover immune tolerance for RA therapy.

To this end, we designed NiH as nanomedicine‐in‐hydrogel composites that allow efficient lymphatic draining and accumulate of cRNPs in LNs, scavenge extracellular/internalized cfDNA, and prolong the release of cGAS inhibitor RU for combinatorial inhibition of proinflammatory responses and enhanced immune tolerance for RA therapy. cRNPs captured extracellular/internalized immunostimulatory DNA while pharmacologically inhibit cGAS, resulting in decreased production of pro‐inflammatory cytokines and expression of costimulatory factors, both of which are essential to present antigens from antigen presenting cells to naïve T cells to elicit antigen‐specific effector T cells.^[^ ^6^, ^45^ ^]^ On the other hand, antigen presentation to T cells without co‐stimulatory signals would induce the apoptosis and deletion of T cells, which would inhibit proinflammatory T cell responses and promote immune tolerance.^[^ ^48^ ^]^

In CIA mouse model, NiH retarded RA progression and reduced arthritis severity, which outperformed control formulations such as cRNPs, likely due to the controlled drug release and prolonged drug retention in immunomodulatory tissues and cells. Flow cytometry analysis revealed that NiH repolarized systemic macrophages toward immunosuppression, as accompanied by reduced production of proinflammatory cytokines. Moreover, NiH decreased the fractions of systemic CD4^+^ T cells and Th17 cells, while increasing systemic immunosuppressive Tregs, MDSCs, as well as M2/M1‐like macrophage ratios. The ability of NiH to elicit systemic immunosuppression and restored immune homeostasis in CIA mice suggests that NiH holds the potential to benefit RA immunotherapy.

## 4 Experimental Section

### Cell Culture

RAW264.7 macrophages were cultured in Dulbecco's modified eagle's medium (DMEM). DC2.4 cells were cultured in RPMI‐1640 medium. All media was supplemented with 10% FBS (Gibco), 100 U mL^−1^ penicillin, and 100 µg mL^−1^ streptomycin. RAW‐Lucia ISG cells were purchased from InvivoGen and cultured using the indicated specifications. All cells were cultured in a humidified atmosphere (5% CO_2_, 37°C).

### Fabrication of cRNPs and NiH

Miktoarm star polymer PGAA was synthesized as reported before.^[^ ^40^ ^]^ Briefly, macroinitiator PEG(‐alkynyl)‐Br was synthesized via a Passerini three‐component reaction. Then PEG(‐alkynyl)‐PDPA and PDMA‐N_3_ were synthesized by atom‐transfer radical‐polymerization. Finally, PEG(‐alkynyl)‐PDPA and PDMA‐N_3_ were conjugated via click chemistry to prepare miktoarm star polymer PGAA, and the structure was confirmed by ^1^H NMR. Next, solvent evaporation was used to fabricate cRNPs. First, PGAA (4 mg) and RU (1 mg; Sigma–Aldrich) mixed in 1 mL tetrahydrofuran (THF) were added dropwise to deionized H_2_O. The mixture was stirred at room temperature overnight to evaporate THF, and then centrifugated (4000 rpm, 5 min) to remove free RU to prepare cRNPs. RU loading in cNPs was measured by high performance liquid chromatograph (HPLC; SHIMADZU), and calculated using formula: (amount of RU in cNPs)/(amount of cRNPs) × 100%, and the drug loading efficiency was calculated using formula: (amount of RU in cNPs)/(amount of feeding RU) × 100%. Empty cNPs were prepared similarly. The size, zeta potential, stability, and morphology of empty cNPs and cRNPs were characterized using Zetasizer (Malvern Nano ZS90) and TEM (JEOL JEM‐1400). Subsequently, 4‐arm PEG‐DBCO, 4‐arm PEG‐azide (MW 20k; Creative PEGWorks) (Scheme S1, Supporting Information), and cRNPs were physically mixed, and further formed hydrogel via DBCO‐azide click reaction for cRNPs encapsulation to prepare NiH. To study drug release, cRNPs and NiH (150 µL) were placed in mini dialysis tubes (MWCO 20000, Thermo Scientific) and immersed in tubes with different release media containing 0.2 wt% Tween 20 (1 mL, pH 5.8 and 7.4, respectively). The above tubes were placed in a shaker (120 rpm, 37°C); then, at a series of time points, 0.1 mL of release medium was sampled and 0.1 mL of fresh release medium was added. The concentrations of RU were measured by HPLC.

### DNA Binding Efficiency

Calf thymus DNA solution (2.5 µL, 1 mg mL^−1^ in PBS) and Ethidium bromide (EtBr) solution (2.5 µL, 1 mg mL^−1^ in PBS) were first mixed. Then different volumes of cNPs were added and the final volumes were adjusted to 120 µL using H_2_O, 12 µL PBS (10×, pH 7.4 and 5.8, respectively), and 0 or 12 µL FBS. After incubation at 37 °C for 12 h, 100 µL supernatant with the remaining DNA/EtBr complexes was transferred to a 96‐well plate. The fluorescence intensity of the complexes was measured on a BioTek Cytation5 plate reader (E_x_: 485 nm, E_m_: 575 nm). The DNA‐binding efficiency with cNPs was calculated using the formula (1 − (*A* − *A*_0_)/(*A*_1_ − *A*_0_)) × 100%, where *A* is the fluorescence intensity of EtBr/DNA complexes of supernatant after adding NPs, *A*_0_ is the EtBr fluorescence intensity, and *A*_1_ is the fluorescence intensity of EtBr/DNA complexes.

### Cell Viability

RAW264.7 cells were seeded in 96‐well plates (0.03 × 10^6^ cells per well) and cultured overnight. cNPs then were added and further cultured for 24 h. Cell viability was measured using an MTS assay (Alfa Aesar).

### The Impact of cRNPs on Cell Immunostimulation by Extracellular/Internalized DNA

To study the impact of cNPs on macrophage inflammatory responses elicited by extracellular DNA, CpG (1 µm) was mixed with cNPs at a series of concentrations. Then the mixture was added to a 96‐well plate seeded with RAW264.7 cells (0.03 × 10^6^ cells per well) and cultured for 24 h. Cell culture medium was collected, and TNF‐α and IL‐6 concentrations were measured using ELISA kits (R&D Systems). Next, to evaluate cNPs inhibition of immunostimulation elicited by internalized DNA in RAW264.7 cells, 1 µm CpG was first added to a 96‐well plate seeded with RAW264.7 cells (0.03 × 10^6^ cells per well) and cultured for 4 h. Then, cells were washed with PBS, and cNPs were added at a series of concentrations. After 24 h, cell culture medium concentrations of TNF‐α and IL‐6 were determined by ELISA (R&D Systems).

### The Impact of cRNPs on the Activation of M1‐like Macrophages

RAW264.7 cells were seeded in 24‐well plates (0.1×10^6^ cells per well) and cultured for 24 h. Cells were incubated with cNPs (20 µg mL^−1^), RU (1 µm), or cRNPs in the presence of CpG (1 µg mL^−1^) and lipofectamine 2000‐transfected Svg3 (100 nm) for 24 h. Cells were harvested for flow cytometry (BD LSRFortessa‐X20) and qPCR (QuantStudio 3 Real‐Time PCR System) analyses. For qPCR analysis, the total RNA was isolated using PureLink RNA Mini Kit (Thermo Fisher), and High‐Capacity cDNA Reverse Transcription Kit (Thermo Fisher) was used for RNA reverse‐transcription. qPCR analysis was performed using Power SYBR Green PCR Master Mix (Thermo Fisher) and primers shown above.

### Cell Uptake

C6 was used as the fluorescent probe. Free C6 and C6/cNPs were prepared as above. RAW264.7 cells were seeded in 12‐well plates (0.2 × 10^6^ cells per well) and cultured overnight. Free C6 or C6/cNPs (1 µg mL^−1^) were added and further cultured for 0.5 and 1 h, respectively. The cells were then harvested, washed, and analyzed using flow cytometry for C6 fluorescence intensity (BD LSRFortessa‐X20). For confocal microscopy, RAW264.7 or DC2.4 cells were seeded in glass dishes and cultured overnight. Then free C6 or C6/cNPs (1 µg mL^−1^) were added and further cultured for 1 h. Cells were washed with PBS, stained with hoechst33342, fixed, and imaged under a confocal microscope (Zeiss LSM 710).

### LN Imaging and RNA‐seq Analysis in Human Subjects

Three RA patients and three healthy controls were included in the study. Ultrasound imaging of their lymph nodes was conducted using a Philips IU Elite ultrasound diagnostic instrument (Philips Healthcare, Seattle, WA) equipped with a L15‐7io probe. The study protocol was approved by the Medical Ethics Committee of Xiangya Hospital Central South University. All subjects signed an informed consent before participation in the study. Published dataset (GSE93776) was used for bioinformatic analysis of bulk RNA‐seq of human PBMCs.^[^ ^39^ ^]^

### Animal Studies

All animal work was in accordance with a protocol (AD10001961) approved by the Institutional Animal Care and Use Committee (IACUC) of Virginia Commonwealth University. Female C57BL/6 mice (6 weeks) were purchased from Charles River Laboratories. Female DBA/1J mice (6 weeks) were purchased from The Jackson Laboratory.

### CIA Mouse Model

The CIA model was established by double immunization of DBA/1J female mice. For the first immunization, mice were injected intradermally at the end of the tail with an emulsion of equal volume bovine type‐II collagen solution (2 mg/mL; Chondrex, Inc.) and complete Freund's adjuvant (Chondrex, Inc.). Twenty‐one days after the first immunization, the above mice received a booster of bovine type‐II collagen solution emulsified in incomplete Freund's adjuvant (Chondrex, Inc.).

### Immune Microenvironment and cGAS Expression in CIA Mice

The CIA mice and age‐matched healthy mice were euthanized, and spleens and inguinal LNs were harvested for immune microenvironment and cGAS expression analysis. Spleens were mechanically dissociated using surgical scissors and strained through 70‐µm cell strainers; LNs were mechanically dissociated using surgical scissors and then treated with collagenase D (1 mg mL^−1^, Sigma) and DNase I (10 U/mL, New England Biolabs) for 10 min at 37 °C. Then the processed LN samples were strained through 70‐µm cell strainers. Harvested dissociated cells were treated with ACK lysis buffer (Gibco). Cells were washed twice with PBS, and collected for qPCR, flow cytometry, and western blot studies.

For qPCR, the total RNA of harvested cells was isolated as described previously. ^[^ ^40^ ^]^ The following forward‐reverse primer pairs (IDT) were used: *Cxcl9* (5'‐CCTAGTGATAAGGAATGCACGATG‐3', 5'‐CTAGGCAGGTTTGATCTCCGTTC‐3'), *Cxcl10* (5'‐ATCATCCCTGCGAGCCTATCCT‐3', 5'‐GACCTTTTTTGGCTAAACGCTTTC‐3'), *Ifnb* (5'‐CGAGCAGAGATCTTCAGGAAC‐3', 5'‐TCACTACCAGTCCCAGAGTC‐3'), *Il6* (5'‐GAGGATACCACTCCCAACAGACC‐3', 5'‐AAGTGCATCATCGTTGTTCATACA‐3'), *IL12* (5'‐CAGAAGCTAACCATCTCCTGGTTTG‐3', 5'‐TCCGGAGTAATTTGGTGCTTCACAC‐3'), *Tnfa* (5'‐GGTGCCTATGTCTCAGCCTCTT‐3', 5'‐GCCATAGAACTGATGAGAGGGAG‐3'), *Mrc1* (5'‐GTTCACCTGGAGTGATGGTTCTC‐3', 5'‐AGGACATGCCAGGGTCACCTTT‐3'), *Gapdh* (5'‐CTTTGTCAAGCTCATTTCCTGG‐3', 5'‐TCTTGCTCAGTGTCCTTGC‐3'), and *Nos2* (5'‐TGCATGGACCAGTATAAGGCAAGC‐3', 5'‐GCTTCTGGTCGATGTCATGAGCAA‐3').

For western blot, cells were lysed with RIPA (Thermo Fisher) buffer supplemented with protease inhibitors (Thermo Fisher). Then the cell lysates were centrifugated at 4 °C (12 000 × *g*, 10 min), and the supernatants were collected. Protein concentration was quantified by the Pierce Micro BCA Protein Assay Kit (Thermo Fisher) and proteins were separated by vertical polyacrylamide gel. After transfer to PVDF membranes, the membranes were blocked with 5% nonfat milk (Fisher Scientific) and then incubated with rabbit anti‐cGAS antibody (Cell Signaling Technology, #31659) and anti‐GAPDH antibody [6C5] (abcam, #ab8245). Gel was visualized using Pierce ECL Plus Western Blotting Substrate (Thermo Fisher), and exposed by the ChemiDoc MP Imaging System (Bio‐Rad).

For flow cytometry, cells were stained with antibodies according to manufacturers’ specifications, and were assessed using a BD LSRFortessa‐X20 cytometer.

### cfDNA Quantification

Extraction of cfDNA from plasma or LNs was performed using Dynabeads SILANE Viral NA kit (Thermo Fisher). For LNs, 50 µL proteinase K (20 mg/mL; Sigma) was added, and LNs were mechanically dissociated using surgical scissors, and then 300 µL lysis/binding buffer was added and mixed prior to cfDNA extraction. The concentration of cfDNA was quantified by Quant‐iT™ PicoGreen® dsDNA reagent and kits (Thermo Fisher).

### Systemic Biosafety Evaluation of cNPs‐H

Mice were injected s.c. with either PBS or cNPs‐H (100 µL; cNPs: 40 mg kg^−1^). Mouse body weight was monitored every 2 days. 6 days later, PBMCs were collected and treated with ACK lysis buffer (Gibco). Cells were washed twice with PBS, collected, and stained with antibodies according to manufacturers’ specifications. Cells were analyzed using flow cytometry (BD LSRFortessa‐X20). Meanwhile, the mice were euthanized, and major organs were harvested and fixed in 10% formalin solution (Sigma) for H&E staining.

### In Vivo Drug Delivery to Draining LNs

DIR (Thermo Fisher) was used as the fluorescent probe for in vivo imaging. DIR/cNPs were prepared as previously described. DIR/cNPs‐H was prepared via the physical mixing of PEG solution and DIR/cNPs. On day 0, 100 µL free DIR, DIR/cNPs, or DIR/cNPs‐H (DIR: 0.5 mg kg^−1^) were s.c. injected at mouse tail base. On days 1, 3, and 5, the DIR fluorescence in mice was imaged by IVIS Spectrum Preclinical In Vivo Imaging System (PerkinElmer IVIS Spectrum). The mice were sacrificed on day 5 for IVIS imaging of the fluorescence in draining LNs and flow cytometric analysis of single cell DIR fluorescence intensity in LN‐residing DCs and microphages (BD LSRFortessa‐X20).

### RA Therapy

CIA mice were randomly divided into five groups, and the treatments began on day 24 after the first immunization. 100 µL PBS, RU‐H, cNPs‐H, cRNPs, or NiH was injected s.c. at mouse tail base every 3 days. The doses of RU and cNPs were 10 and 35 mg kg^−1^, respectively. Mice were monitored every 3 days and the clinical score recorded using the following the scoring criteria: score 0: normal; score 1: mild, but definite redness and swelling of the ankle or wrist, or apparent redness and swelling limited to individual digits, regardless of the number of affected digits; score 2: moderate redness and swelling of ankle or wrist; score 3: severe redness and swelling of the entire paw including digits; and score 4: maximally inflamed limb with involvement of multiple joints. On day 43, peripheral blood and spleens were collected. Peripheral blood was centrifuged at 800 × *g* for 5 min at 4°C to obtain the PBMCs and plasma for cytokine test and cfDNA extraction. Spleens were mechanically dissociated by surgical scissors and strained through a 70‐µm cell strainer. Harvested splenocytes and PBMCs were then processed as described above. Cells were analyzed using flow cytometry (BD LSRFortessa‐X20).

### Flow Cytometry

Anti‐mouse antibodies were obtained from Biolegend and used according to manufacturers’ specifications: CD45 (Cat No. 103133 and 103108), CD3 (Cat No. 100210), CD4 (Cat No. 100434 and 100446), CD8α (Cat No. 100714), CD86 (Cat No. 105008 and 105026), CD11b (Cat No. 101210), CD206 (Cat No. 141721), CD25 (Cat No. 101904), CD80 (Cat No. 104718), NOS2 (Cat No. 696804), CD11c (Cat No. 117346), F4/80 (Cat No. 123118), Ly‐6G/Ly‐6C (Cat No. 108417), FOXP3 (Cat No. 126408), IL‐17A (Cat No. 506940), CD16/32 (Cat No. 101320). Flow cytometry data were analyzed using FlowJo software.

### Statistical Analysis

Data were presented as mean ± standard deviation (SD) and mean ± standard error of the mean (s.e.m). Statistical significance was evaluated by one‐way or two‐way ANOVA when experimental groups were compared (Microsoft Excel).

## Conflict of Interest

F.C. and G.Z. were listed as inventors for a related patent application.

## Supporting information

Supporting Information

## Data Availability

The data that support the findings of this study are available from the corresponding author upon reasonable request.
