# Supplementary material for: Targeting Lymph Nodes for Systemic Immunosuppression Using Cell‐Free‐DNA‐Scavenging And cGAS‐Inhibiting Nanomedicine‐In‐Hydrogel for Rheumatoid Arthritis Immunotherapy
Source: Adv Sci (Weinh). 2023 Jul 12;10(26):2302575. doi: 10.1002/advs.202302575 (PMC10502670; doi:10.1002/advs.202302575)
Supplement: Supplementary file 1 — Supporting Information [file ADVS-10-2302575-s001.pdf]

ADVANCED SCIENCE
Open Access

Supporting Information

for *Adv. Sci.*, DOI 10.1002/advs.202302575

Targeting Lymph Nodes for Systemic Immunosuppression Using Cell-Free-DNA-Scavenging And cGAS-Inhibiting Nanomedicine-In-Hydrogel for Rheumatoid Arthritis Immunotherapy

Furong Cheng, Ting Su, Yangtengyu Liu, Shurong Zhou, Jialong Qi, Weisheng Guo and Guizhi Zhu**

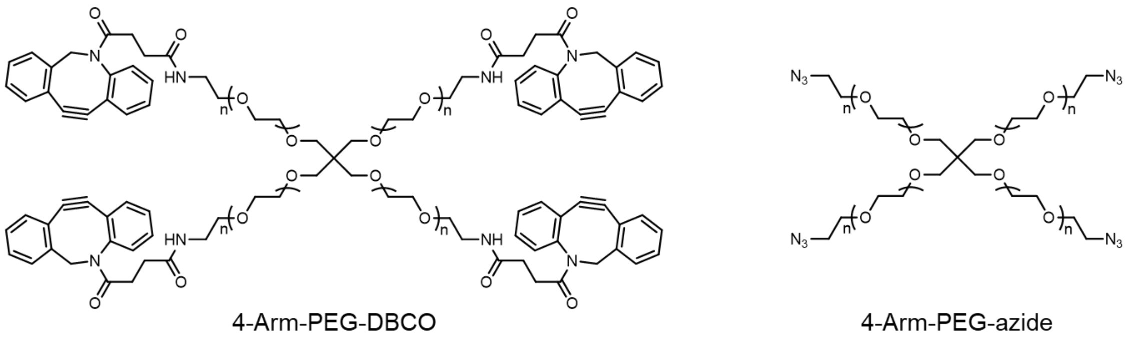

Scheme S1. Structures of 4-arm-PEG-DBCO and 4-arm-PEG-azide.

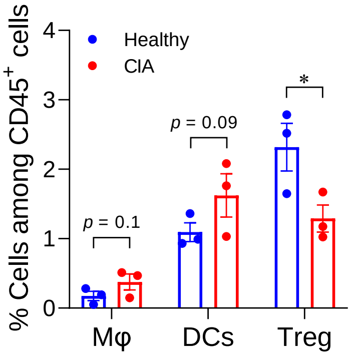

Figure S1. Flow analysis of immune cell subsets in LNs from healthy and CIA mice. $n = 3$. Data: mean ± s.e.m. P values were determined by one-way ANOVA, followed by Tukey's multiple comparison test (*$p < 0.05$).

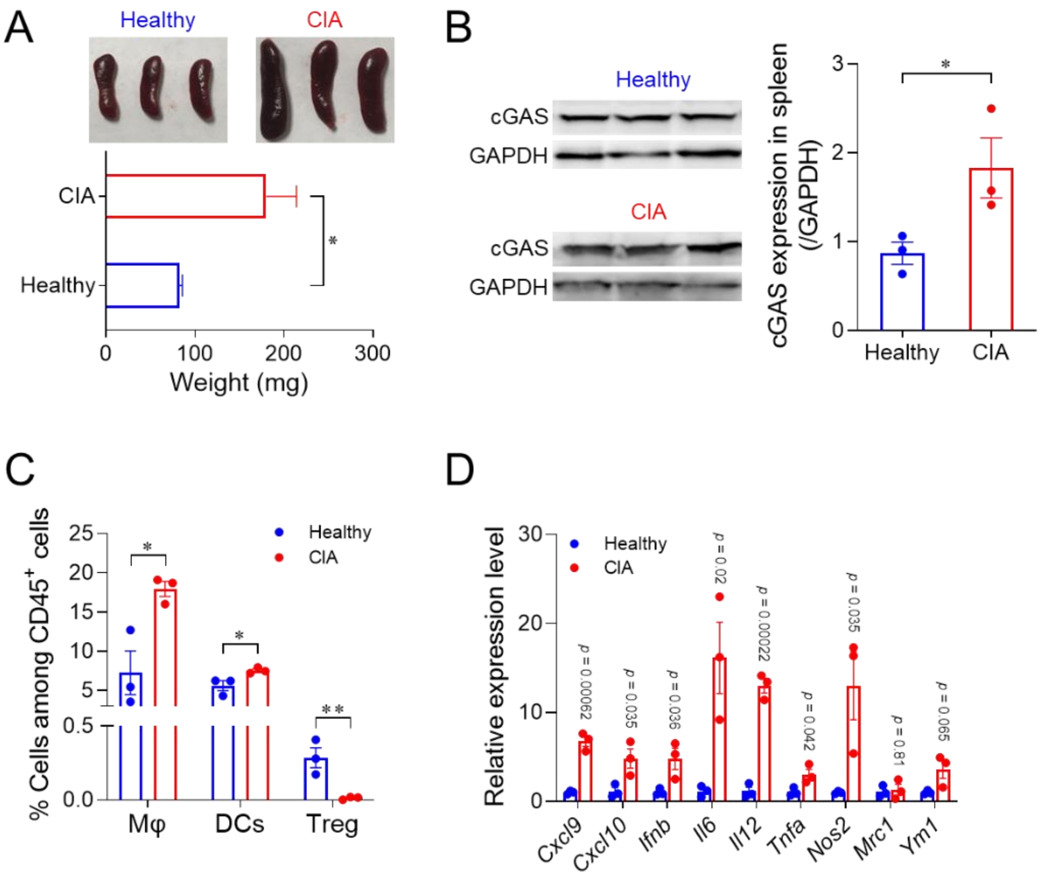

Figure S2. The spleens of CIA mice are highly proinflammatory with abundant cfDNA and upregulated cGAS. Photographs and weights (**A**) of spleens from healthy and CIA mice. (**B**) Western blot assay of cGAS expression in spleen from healthy and CIA mice. (**C**) Flow cytometric analysis of the splenic immune microenvironment of healthy and CIA mice. (**D**) qPCR results for cytokines and chemokines in spleen of healthy and CIA mice. Data: mean ± s.e.m. $n = 3$. P values were determined by the one-way (**B**, **C**) or two-way (**A**, **D**) ANOVA followed by Tukey's multiple comparison test (*$p < 0.05$; **$p < 0.01$).

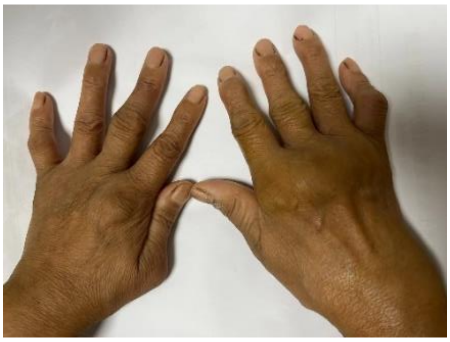

Figure S3. Representative photograph of a RA human patient.

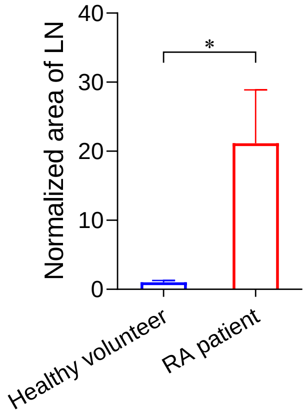

Figure S4. The normalized area of LNs measured from ultrasound images. The area of LN was measured by ImageJ software. n = 3. Data: mean ± s.e.m. P values were determined by one-way ANOVA, followed by Tukey's multiple comparison test (*p < 0.05).

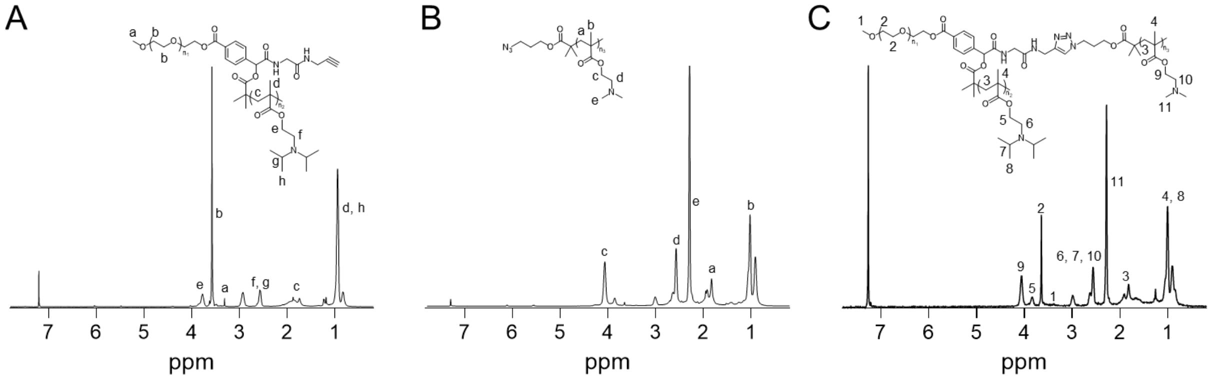

Figure S5. ^{1}H NMR ($CDCl_3$-*d*) spectra of PEG-PDPA (**A**), PDMA (**B**), and PEG−PDMA−PDPA (**C**).

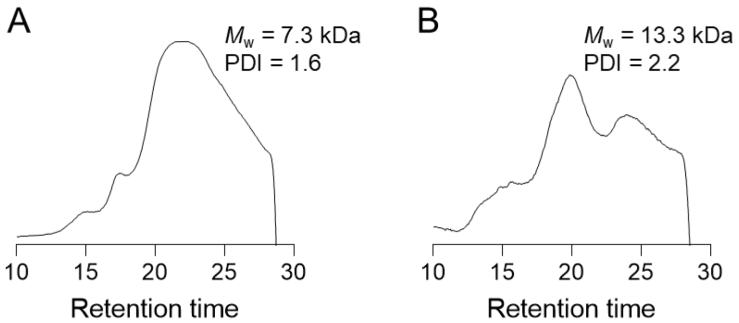

Figure S6. GPC spectra of PDMA (**A**) and PEG−PDMA−PDPA (**B**). N,N-Dimethylformamide was used as the eluent (1 mL/min); and the molecular weight (M_w) and polymer dispersity index (PDI) were obtained with polyethylene glycol as the standard.

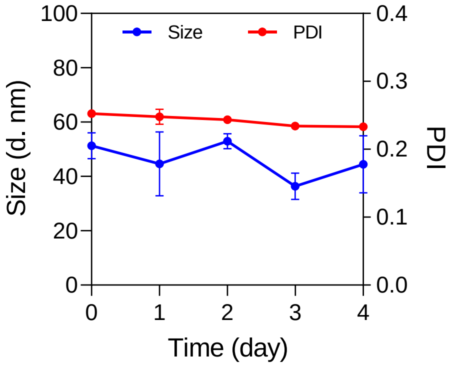

Figure S7. The hydrodynamic diameters of cNPs stored in water over 4 days, indicating their good stability (1 mg/mL; ambient temperature; n = 3). Data: mean ± SD.

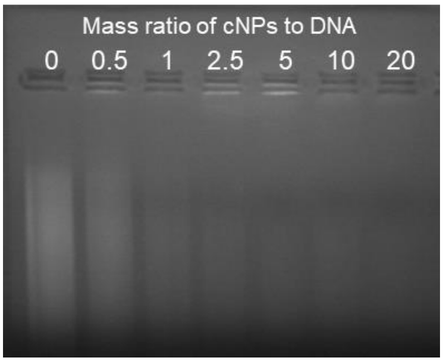

Figure S8. Gel retardation assay showing the DNA binding with cNPs at a series of cNPs: DNA mass ratios.

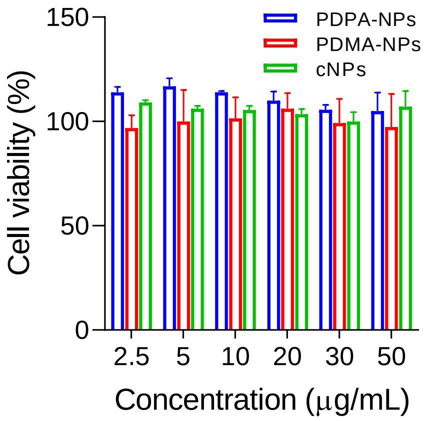

Figure S9. MTS assay results showing the cell viability of NPs in RAW264.7 murine macrophages. Treatment time: 24 h. n = 5. Data: mean ± SD.

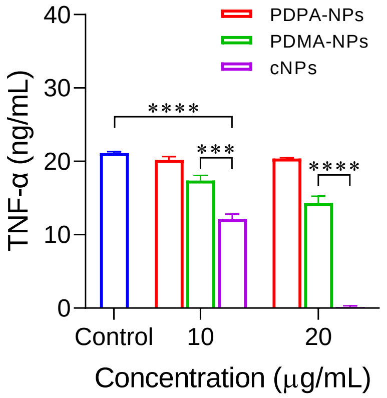

Figure S10. cNPs inhibited TNF-α expression in RAW264.7 cell. RAW264.7 cells were incubated with 1 μM CpG and cNPs (10 and 20 μg/mL) at 37 °C for 24 h. Medium TNF-α concentrations were measured by ELISA. $n = 3$. Data: mean ± SD. P values were determined by two-way ANOVA, followed by Tukey's multiple comparison test (***$p < 0.001$, ****$p < 0.0001$).

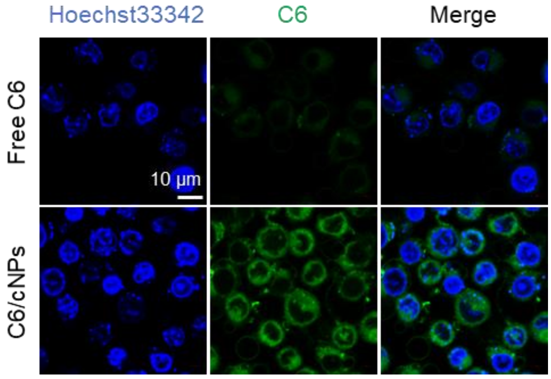

Figure S11. Confocal microscopy images showing the intracellular delivery of C6 by cNPs into DCs (treatment: 1 h).

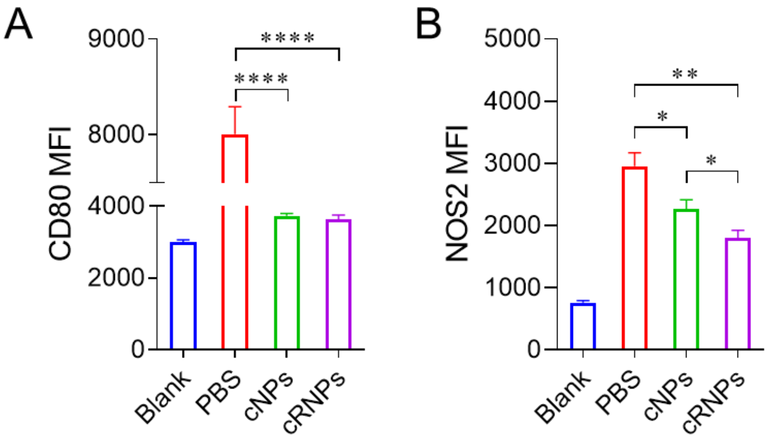

Figure S12. MFI of the CD80 (**A**) and NOS2 (**B**) on RAW264.7 cells after incubation with cRNPs for 24 h (n = 3). RAW264.7 cells were incubated with cRNPs (RU: 2 μM; cNPs: 20 μg/mL) and 1 μM CpG + 100 nM Svg3 (transfected by Lipofectamine 2000) at 37 °C for 24 h. n = 3. Data: mean ± SD. *P* values were determined by two-way ANOVA, followed by Tukey's multiple comparison test (**p* < 0.05, ***p* < 0.01, *****p* < 0.0001).

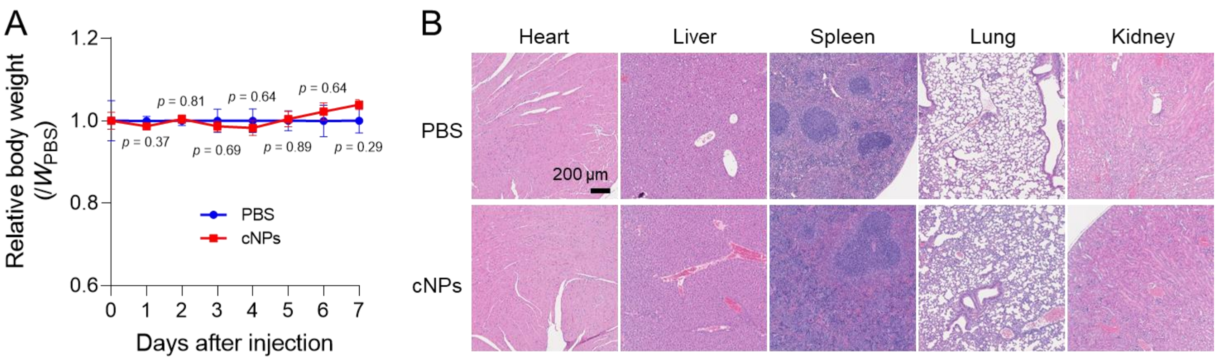

Figure S13. (**A**) Relative mouse body weights after *s.c.* injection of cNPs and PBS control (cNPs: 40 mg/kg). cNPs or PBS were *s.c.* injected on day 0. C57BL/6 mice were sacrificed on day 7, and main organs were harvested for H&E staining. n = 3. Data: mean ± s.e.m. *P* values were determined by two-way ANOVA. (**B**) Representative H&E staining images of major organs isolated on day 7.

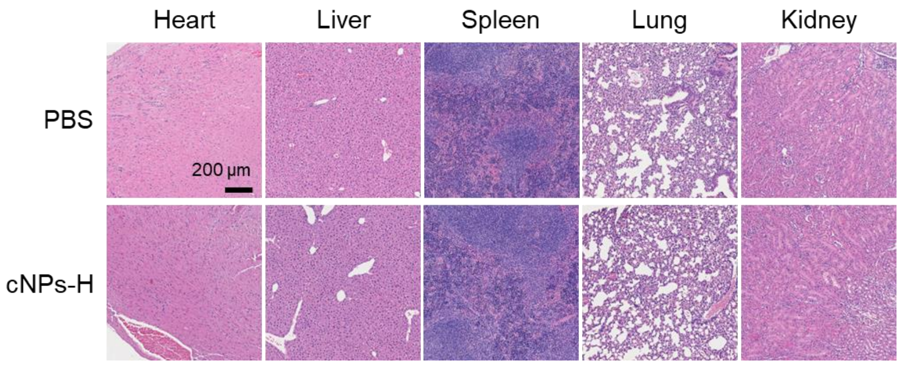

Figure S14. Representative H&E staining images of major organs of C57BL/6 mice isolated 6 days following administration with cNPs-H and PBS, respectively.

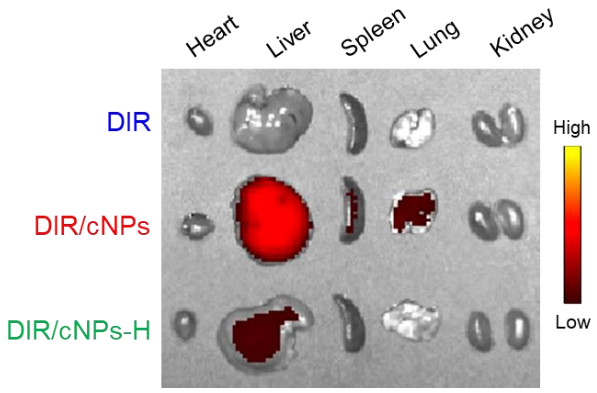

Figure S15. IVIS images of *ex vivo* major organs collected 5 days post administration.

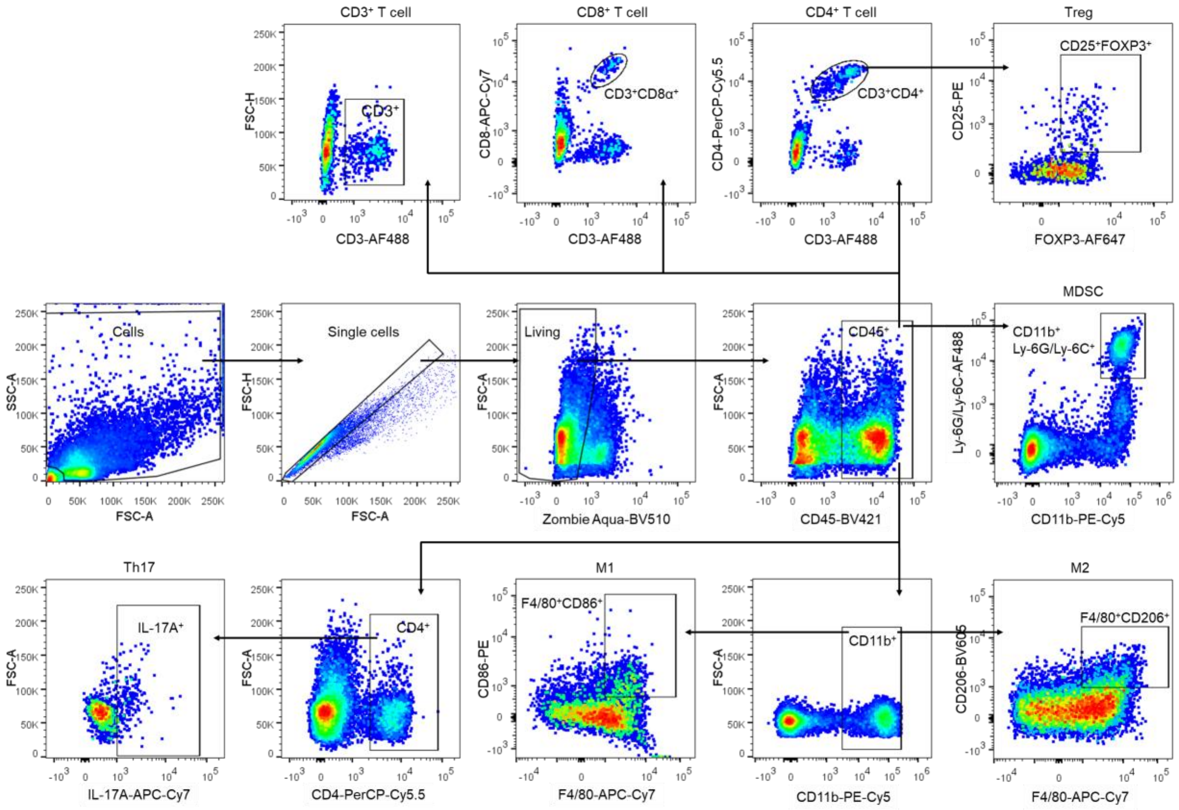

Figure S16. Representative gating tree used for immune analysis.

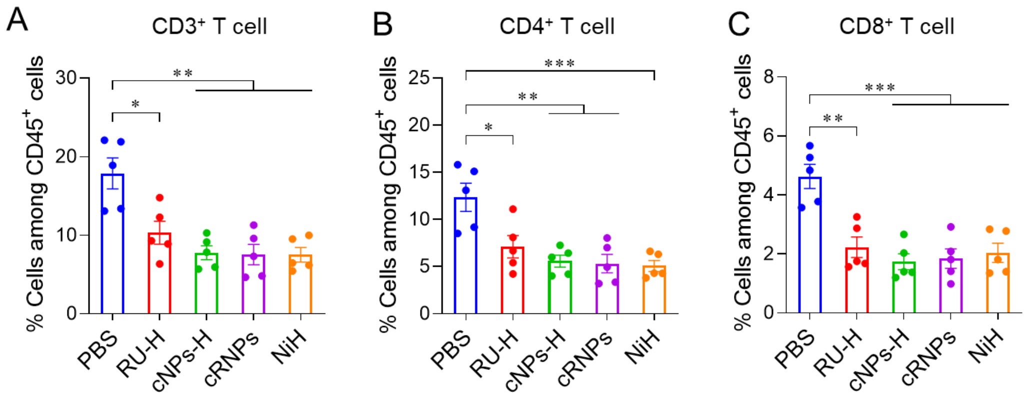

Figure S17. PBMC immune cell analysis from CIA mice treated as indicated (day 43). Shown are the flow cytometric quantification of the fractions of PBMC CD3$^+$ T cells (**A**), CD4$^+$ T cells (**B**), and CD8$^+$ T cells (**C**) among total CD45$^+$ cells. n = 5. Data: mean ± s.e.m. *P* values were determined by one-way (**B**, **C**) or two-way (**A**) ANOVA, followed by Tukey's multiple comparison test (*p < 0.05, **p < 0.01, ***p < 0.001).

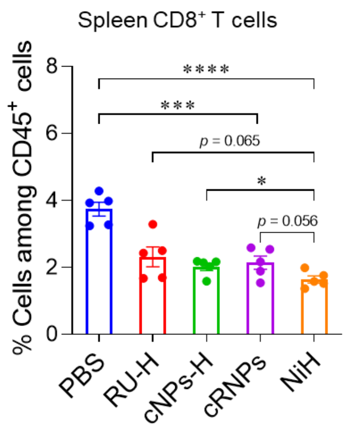

Figure S18. Flow cytometric quantification of splenic CD8$^+$ T cells among total CD45$^+$ cells from as-treated CIA mice (day 43). n = 5. Data: mean ± s.e.m. P values were determined by two-way ANOVA, followed by Tukey's multiple comparison test (*$p < 0.05$, ***$p < 0.001$, ****$p < 0.0001$).

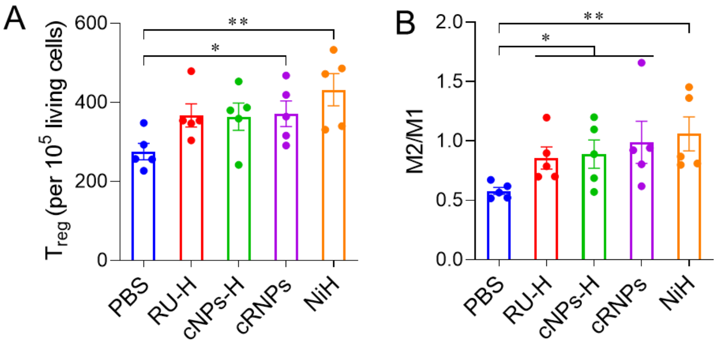

Figure S19. Population of Treg cells (**A**) and M2/M1-like macrophage ratio (**B**) in among total PBMC CD45$^+$ cells (Day 43). n = 5. Data: mean ± s.e.m. P values were determined by one-way (**B**) or two-way (**A**) ANOVA, followed by Tukey's multiple comparison test (*p < 0.05, **$p < 0.01$).

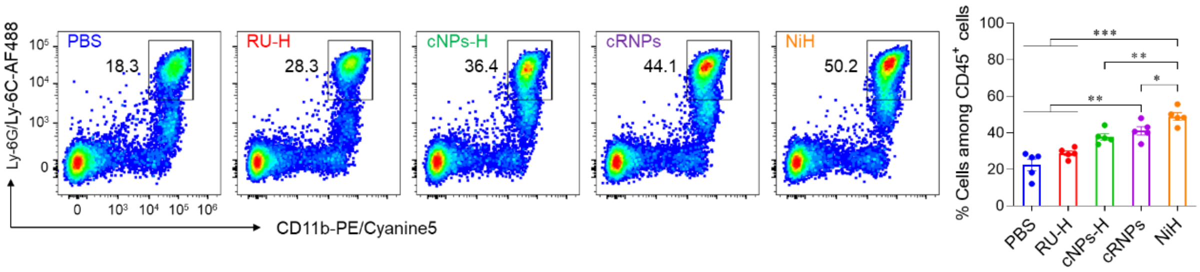

Figure S20. Representative flow cytometry plots (left) and the fractions of PBMC MDSCs among total CD45$^+$ cells (day 43). n = 5. Data: mean ± s.e.m. P values were determined by two-way ANOVA, followed by Tukey's multiple comparison test (*$p < 0.05$, **$p < 0.01$, ***$p < 0.001$).
